# Avoiding URL Reference Degradation in Scientific Publications

**DOI:** 10.1371/journal.pbio.0020099

**Published:** 2004-04-13

**Authors:** Desiree P Kelly, Eric J Hester, Kathryn R Johnson, Lauren F Heilig, Amanda L Drake, Lisa M Schilling, Robert P Dellavalle

## Abstract

Arguments are presented concerning the deposit of Internet-based information into the Internet Archive, a digital library of Internet sites and other digital data

While we applaud PloS' use of Digital Object Identifiers (“The What and Whys of DOIs,” PLoS Biol 1: e57 doi: 10.1371/journal.pbio.0000057), we also note the lack of provisions in your instructions for authors for preserving access to electronic information residing at a cited Internet addresses via Uniform Resource Locators (URLs). Medical and scientific literature increasingly cites information only found on the Internet. However, URLs may become inaccessible shortly after article publication.

Please consider requiring PLoS' authors to (1) submit all cited URLs to the Internet Archive (www.archive.org), a nonprofit organization that has been preserving electronic content since 1996, and (2) maintain a printed copy of the electronic information for future communication until the URL becomes available at the Internet Archive (about a six-month lag time).

The Internet Archive, the largest digital library of Internet sites and other digital data, stores cited Internet information at no cost to the author, reader, or publisher. By requiring PLoS' authors to submit all cited Internet-based information to the Internet Archive, PLoS will better preserve the integrity of its content for the future.

## PLoS' Response

Ms. Kelly and colleagues raise an important issue about the ephemeral nature of many information sources on the Internet. In the case of online scholarly literature, information is more likely to be archived and able to be found—indeed, an open-access article is one in which, according to the Bethesda Definition, “A complete version of the work and all supplemental materials, including a copy of the permission…, in a suitable standard electronic format is deposited immediately upon initial publication in at least one online repository that is supported by an academic institution, scholarly society, government agency, or other well-established organization that seeks to enable open access, unrestricted distribution, interoperability, and long-term archiving (for the biomedical sciences, PubMed Central is such a repository).”

Other types of Internet-based information are more likely to change, move, or be removed. We agree that wherever possible we must find a way to preserve the relevant information from the sources cited in our articles.

PLoS has always encouraged authors to submit supporting information for their research articles, including raw datasets, spreadsheets, multimedia, and snapshots of Web-based interactive tools. PLoS makes this supporting information available to everyone for download and use. PLoS also requires authors to deposit all appropriate datasets, images, and information in public databases and to list the relevant accession and version numbers in the article.

The question, then, is how PLoS and its authors can preserve access to other Internet-based information, including organizational Web sites, articles in the popular media, or interactive databases.

Although submitting cited URLs to the Internet Archive is worthwhile, it is still (unfortunately) far from ideal. The Internet Archive is best at archiving simple HTML and may be the most appropriate place to archive a Web site an author has cited for its static information content. The Internet Archive does not, however, archive content with password restrictions or “crawling” restrictions, and it allows the removal of already archived content at the request of Web administrators; it would therefore not be an effective archive, for example, for popular press articles that have restricted access. In addition, the Internet Archive cannot preserve functions that interact with the originating server, so it is not an appropriate way to archive a Web site an author has cited, for example, for its useful interactive tools. Finally, there is currently no automated way for publishers to redirect links from the original address to the address on the Internet Archive.

For the time being, PLoS plans to review all electronic citations on a case-by-case basis and, when appropriate, request that authors submit the cited Web site URL to the Internet Archive and additionally submit a digital copy of the information to PLoS for internal archiving.

We would also like to encourage further input on this issue from the scientific and medical community and urge them to support the Internet Archive and other organizations working to preserve the digital record for future generations.

